# Editorial: Mitochondrial Proteomics: Understanding Mitochondria Function and Dysfunction Through the Characterization of Their Proteome

**DOI:** 10.3389/fcell.2020.608753

**Published:** 2020-12-10

**Authors:** Alberto Ferri, Pablo M. Garcia-Roves, Luisa Pieroni

**Affiliations:** ^1^Department of Experimental Neuroscience, Istituto di Ricovero e Cura a Carattere Scientifico-S.Lucia Foundation, Rome, Italy; ^2^Department of Biomedical Science, Institute of Translational Pharmacology, Italian National Research Council, Rome, Italy; ^3^Department of Physiological Sciences, University of Barcelona and Bellvitge Biomedical Research Institute (IDIBELL), Barcelona, Spain; ^4^Centro de Investigación Biomédica en Red de Fisiopatología de la Obesidad y la Nutrición (CIBEROBN), Instituto de Salud Carlos III, Madrid, Spain

**Keywords:** mitochondria, proteome, post-translational modifications, metabolism, neurodegeneration

The development of OMICS sciences, and in particular Proteomics, has provided new tools to investigate the mitochondrial role in health and disease on a system biology scale.

This Research Topic aims to collect papers describing how evidence based on proteomics data can contribute to gaining a deep understanding of mitochondrial structure and function and to unveil unknown or multifactorial features of mitochondrial physiological function and dysfunction.

This exciting editorial initiative has allowed us to collect seven interesting contributions.

Three original research articles investigate mitochondrial proteome changes in response to non-physiological stimuli, such as micro-environmental modifications or neurological diseases.

The paper by Maffioli et al. sheds light on mechanotransduction, a particular ability of cells to respond and adapt their structure and function to biophysical changes in their microenvironment. The authors studied this phenomenon in cellular models of rodent pancreatic β cells cultured on artificial microenvironment, a nanostructured surface assembled by Supersonic Cluster Beam Deposition of zirconia nanoparticles. Following their previous study, they used mass spectrometry-based proteomics to investigate the involvement of mitochondria. They showed that mitochondria morphology and proteome can be regulated by nanotopography leading to a metabolic switch in these cells.

Parkinson Disease is the topic of the research article by Zilocchi et al., which describe a proteomic study aimed to unravel mitochondrial and whole cell proteome alteration in human skin fibroblasts of *PARK2*-mutated patients. Mitochondrial function is involved in a series of molecular pathways related to Parkinson Disease mechanisms. The Park2-mutated model investigated in this article offers a unique background of mitophagy impairment hence the characterization of mitochondrial proteome was particularly intriguing to understand tentative causative relationships at the base of this disease. The quantitative proteomic analysis coupled with network-based system biology studies allowed these authors to highlight important molecular pathways altered by Park protein loss of function, particularly those related to the unfolded protein response and GTPases mediated signal transduction.

Gella et al., with an elegant and complex experimental design, address the issue of understanding mitochondrial dysfunctions in Leigh Syndrome (LS), one of the most severe neuromuscular pathologies belonging to the category of the Mitochondrial Diseases (MDs). The lack of NDUFS4, one of the assembly subunits of Complex I, determines the development of LS in humans. The authors based their research on the hypothesis that MDs are characterized by an anatomical and cellular specificity. By means of viral vectors, capable of targeting mitochondria in specific mouse tissues, they were able to isolate NDFUS4-deficient mitochondria from glutamatergic neurons of the vestibular nucleus in the brain of a mouse model of the human pathology. The analysis of the whole mitochondrial proteome and acetylome with sophisticated MS-based experiments allowed them to investigate specifically the neuronal susceptibility to mitochondrial dysfunction showing that, in NDUFS4-depleted mice, the whole complex I assembly and function are affected. Furthermore, they highlighted the role of other mitochondrial and non-mitochondrial proteins whose expression and/or acetylation was altered by the pathology (e.g., PHD, PKCδ, complement factor C1q, or complex V).

Combining mouse genetics and cell-type-specific mitochondria isolation with state-of-the-art mass-spectrometry-based proteomics, these authors collected observations otherwise impossible in a global analysis of a whole cell proteome, which is perfectly in line with the aim of this Research Topic.

The articles collection in this Research Topic has been enriched by two very interesting Review papers.

Reina et al. focused their attention on PTMs occurring on mitochondrial proteins and the functional consequence of these modifications on the mitochondria oxidative metabolism. In particular, they reviewed the most recent research reporting on cysteine oxidative modifications of the three VDAC isoforms, the voltage-dependent anion selective channel—a particularly abundant protein on the mitochondrial outer membrane. Mass-spectrometry-based datasets were fundamental to map those specific PTMs.

An interesting review by Refolo et al. sheds light on the unconventional function of mitochondria as a pivotal organelle controlling signaling pathways to respond to external stimuli, such as a viral infection, mounting an adaptive response or inducing cell death. In this article, the authors point their attention on the role of mitochondrial antiviral signaling (MAVS) protein, and they thoroughly reviewed up-to-date literature describing MAVS signalosome assembly and recruitment in a host cell upon an RNA virus infection. In compliance with this Research Topic, they reviewed with particular interest those articles describing how mass spectrometry based mitochondrial proteomics approaches contribute to the comprehension of complicated mechanisms allowing the dissection of important protein–protein interactions or PTMs involving MAVS. This mechanism has found particular interest in the recent investigations on SARS-CoV-2 replication molecular features.

A glance at the fundamental technological update in this field could not be missed, and two methodological research articles have thus been published.

Van Esveld et al. looked at mitochondrial proteomes from a different perspective, approaching the characterization of proteins whose function is required to regulate the gene expression system of mitochondrial proteins encoded by both mtDNA and nDNA, particularly focusing on the poliA-RNA-interacting proteins. They described an effective procedure to enrich mitochondrial poliA-RNA-binding proteins to be analyzed by means of a Mass-Spectrometry-based experiment. They integrated data collected with this experimental setup with whole-cell poliA-RNA-interacting proteome datasets previously published to rank the complete human proteome for the likelihood of mtRNA interaction.

Marini et al. focused their interest on the implementation of an experimental workflow that combines biochemistry and mass spectrometry to pursue a comprehensive definition of mitochondrial proteome and possibly mine mitochondrial uncharacterized proteins. In this brief research report article, a deep analysis of the mitochondrial proteome was performed, exploiting cells and organelle sub-proteome approach, in order to reduce sample complexity and focus the analysis specifically on mitochondria and sub-mitochondria compartments. Moreover, a multiple proteolytic enzyme approach and two different mass spectrometry shotgun experimental setups allowed the authors to draw a detailed picture of the mitochondrial proteome, including a small group of proteins whose functions was not annotated yet defined as *dark proteins*. This work demonstrates how proteomics can provide interesting information to contribute to molecular medicine and pharmaceutical research, potentially mapping new lead compound targets.

## Author Contributions

All authors listed have made a substantial, direct and intellectual contribution to the work, and approved it for publication.

## Conflict of Interest

The authors declare that the research was conducted in the absence of any commercial or financial relationships that could be construed as a potential conflict of interest.

